# Methylation of HPV16 and *EPB41L3* in oral gargles and the detection of early and late oropharyngeal cancer

**DOI:** 10.1002/cam4.4757

**Published:** 2022-05-26

**Authors:** Brittney L. Dickey, Belinda Nedjai, Matthew D. Preece, Michael J. Schell, David Boulware, Junmin Whiting, Bradley Sirak, Martha Abrahamsen, Kimberly A. Isaacs‐Soriano, Kayoko Kennedy, Christine H. Chung, Anna R. Giuliano

**Affiliations:** ^1^ Center for Immunization and Infection Research in Cancer H. Lee Moffitt Cancer Center and Research Institute Tampa Florida USA; ^2^ Department of Cancer Epidemiology H. Lee Moffitt Cancer Center and Research Institute Tampa Florida USA; ^3^ Centre for Cancer Prevention Wolfson Institute of Preventive Medicine, Queen Mary University London UK; ^4^ Department of Biostatistics and Bioinformatics H. Lee Moffitt Cancer and Research Institute Tampa Florida USA; ^5^ Department of Head and Neck‐Endocrine Oncology H. Lee Moffitt Cancer and Research Institute Tampa Florida USA

**Keywords:** early detection, HPV, methylation, oropharyngeal cancer

## Abstract

As oropharyngeal cancer (OPC) associated with human papillomavirus (HPV) increases in men, the need for a screening test to diagnose OPC early is crucial. While HPV‐associated OPC has a favorable prognosis, recurrence is likely, and metastatic OPC is often incurable regardless of HPV status. Our previous study of pretreatment, male OPC cases (*n* = 101) and age‐ and smoking‐matched controls (*n* = 101) found methylation of the host *EPB41L3* tumor suppressor gene and HPV16 in the oral gargle was correlated with these biomarkers in the tumor. Methylation of these genes in the oral gargle was significantly (*p* < 0.0001) higher among cases compared to controls. To further study the utility of HPV16/*EPB41L3* methylation, we expanded the sample size and specifically increased the number of early OPC cases (T1‐T2, N0‐N1; small tumors with a single ipsilateral node <3 cm) to evaluate these biomarkers in early and late OPC. This study included 228 OPC cases, 92 of which were early cases and frequency matched to 142 healthy controls. In logistic regression, the AUC for HPV16/*EPB41L3* methylation for all OPC cases was 0.82. Among early and late OPC cases, the AUC was 0.78 and 0.85, respectively. For early cases, 76% sensitivity was achieved, replicating results from our prior study, with a specificity of 65%, indicating room for improvement. The ability of HPV16/*EPB41L3* methylation to distinguish OPC from healthy controls highlights its utility as a potential biomarker for OPC. However, the inability to predict early OPC better than late stage OPC indicates the need for additional biomarkers to improve screening performance.

## Graphical abstract

We have expanded our previous study with priority in increasing early OPC case sample size to specifically test the performance of HPV16/EPB41L3 methylation as a biomarker for early OPC detection. This study, like the prior study, found HPV16/EPB41L3 methylation as a marker for OPC. Additionally, it has shown the potential for HPV16 and EPB41L3 methylation as a key factor in early detection of OPC but with more biomarkers needed to optimize detection for early OPC.

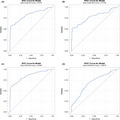

## 1 INTRODUCTION

Oropharyngeal cancer (OPC) incidence is rising in men in the United States, with more cases attributed to human papillomavirus (HPV) infection than other previously recognized factors such as smoking and alcohol.^1^ HPV‐associated OPC is up to fivefold higher among men compared to women.^2^, ^3^

As HPV‐associated OPC rates have increased, it has become clear that these cases have different disease prognosis and survival compared to OPC cases associated with lifestyle factors only. In particular, HPV‐associated OPC has better treatment outcomes, though recurrence remains between 13 and 25% within 2 years.^1^, ^4^ Regardless of HPV association, OPC tumors tend to require extensive treatment including potentially disfiguring surgery and radiation associated with physical and functional side effects such as dysphagia, all further exacerbated by the mental anguish associated with visible scars and deformities.^5^, ^6^

Unlike other HPV‐associated cancers, there is no method to screen for precancerous lesions or to detect the tumors early. Detection of early OPC (T1‐T2, N0‐N1; small tumors with only a single ipsilateral positive node <3 cm) lessens the extent of treatment and reduces the associated adverse consequences.^7^ As such the goal has been to develop a biomarker panel that can detect early OPC. Previously, biomarkers for head and neck cancer have been detected with limited efficacy and imaging and cytology was tested with little to no utility.^8^, ^9^, ^10^, ^11^, ^12^ In a prior study from our group, we demonstrated that methylation of oral gargle and tumor HPV16 L1, L2, and E2 and *EPB41L3* were strongly associated.^13^ In addition, these markers detected in the oral gargle were associated with OPC detection in case control analyses. However, as that study only included 19 early OPC cases, we were unable to reliably assess the utility of these methylation biomarkers for the detection of early OPC. In the current study, we replicate the investigation of oral gargle HPV16 and host gene methylation with a larger overall sample size and increased number of early OPC cases.

## 2 MATERIALS AND METHODS

### 2.1 Study population

Male OPC cases were recruited from May 2014 to March 2020 from the Moffitt Cancer Center Head and Neck Cancer Radiation Oncology and Senior Adult Oncology clinics. Medical record review identified potential study participants. Once determined eligible, a clinical coordinator approached participants to initiate study enrollment. Interested and eligible cases then signed an informed consent prior to any study procedures. Study eligibility criteria were as follows: male 18 years and older, with a new, histologically confirmed oropharyngeal squamous cell carcinoma (C01.9 base of tongue; C05.1 soft palate, not otherwise specified [NOS]; C05.2 uvula; C09.0 tonsillar fossa; C09.1 tonsillar pillar; C09.8 overlapping lesion of the tonsil; C09.9 tonsil, NOS; C10.0 vallecula; C10.2 lateral wall of epiglottis; C10.3 posterior wall of epiglottis; C10.8 overlapping lesion of oropharynx and C10.9 oropharynx, NOS.) Cases who had received treatment prior to enrollment or did not complete the study survey were excluded. Advarra Institutional Review Board and the Moffitt Cancer Center Scientific Review Committee approved these studies.

Cancer‐free controls were selected from the US participants of the HPV Infection in Men (HIM) Study which recruited 18‐ to 70‐year‐old men, with no history of an HPV‐related cancers, HPV vaccine, or HIV/AIDS. Controls were frequency matched by age within 5 years and smoking status (never, former, and current).

### 2.2 Data collection

Participants in both studies provided an oral gargle specimen in which they gargled mouthwash for 30 seconds before dispensing it into a 50‐ml conical tube. Specimens were centrifuged at 2000*g* for 15 min. The cell pellet was washed three times in 20 ml of cold phosphate‐buffered solution (PBS), inversion mixed to assure thorough homogenization, and then centrifuged at 2000*g* for 15 min at 4°C. The remaining cell pellet was resuspended in 1.2 ml PBS and stored at −80°C. Oral HPV DNA was extracted using the automated BioRobot MDx (Qiagen). HPV status for both cases and controls was obtained using the HPV SPF_10_ PCR‐DEIA‐LiPA_25_ line probe assay (DDL Diagnostic Laboratory, Rijswik, the Netherlands).^14^

Cases and controls completed a computer‐assisted risk survey which questions relating to demographic characteristics, personal and family cancer history, oral health, sexual behavior, alcohol, tobacco, and other drug use.

### 2.3 HPV and host gene methylation

*EPB41L3* and HPV gene methylation were conducted as described in our previous study.^13^ In short, samples were tested for methylation of three CpG sites (438, 427, and 425) in the tumor suppressor gene, *EPB41L3,* using PyroMark. The viral methylation status of the CpG sites in the L1 (6367, 6389), L2 (4257, 4262, 4266, 4269, 4275, 4282) and E2 (3412, 3415, 3417, 3433, 3436) regions of HPV16 determined by pyrosequencing specimens were found to be positive for HPV16. Bisulfite conversion reactions were conducted on 200 ng of DNA using the EZ DNA methylation kit (Zymo Research, Irvine, CA). Converted DNA was then purified and amplified by PCR primers using a converted DNA equivalent of 1500 cells and the Pyro‐Mark PCR kit (Qiagen). Primers were designed with short amplicons (90–140 base pairs each) using the PyroMark Assay Design software (V2.0.1.15 Qiagen). Streptavidin beads (GE Healthcare, Buckinghamshire, UK) were used to capture the products of PCR in 96‐well plates. They were then pyrosequenced using PyroGold reagents and a PyroMark TMQ96 ID (Qiagen) instrument. A standard curve was used as a positive control of 0, 50, and 100% methylated DNA along with a non‐template control.

### 2.4 Statistical analyses

Analyses in our prior study were also used for this study as needed.^13^ First, demographics, sexual behavior, oral health, and oral gargle HPV status were compared for cases (*n* = 228) and controls (*n* = 142) using the Cochran–Mantel–Haenszel (CMH) exact test. Mean value of *EPB41L3* methylation among early disease (T1‐2 N0‐1 [small tumors with only a single ipsilateral positive node <3 cm]) and late disease OPC was compared between cases and controls using the Kruskal–Wallis test with a post hoc Dunn's test for pairwise comparisons.

As described in our prior study, oral HPV16 alone does not predict case status, and its performance is worse among younger and early disease cases, the target population of early screening tests.^13^ However, oral HPV16 methylation occurs rarely in the absence of cancer. The previous study included 101 cases (19 early and 82 late disease) and 101 controls. For this study, we expanded the sample size with the priority to increase early OPC cases. Cases and controls included in the previous study were batch‐adjusted by multiplying the values by 1.2 per our examination of batch effect using weighted median ratio from the controls, early cases, and late cases.

Logistic regression was performed on the combined oral *EPB41L3* and HPV16 methylation data by giving all CpG sites equal weight and HPV genes (L1, L2, and E2) dummy coded as 1, if present. Receiver operating characteristic (ROC) curves were created for the combined HPV16 gene and *EPB41L3* methylation levels with area under the curve (AUC) generated by the Wilcoxon test. Methylation cut points were identified using Youden's J (J statistic) and were selected with a goal of maximizing specificity while maintaining a sensitivity greater than 70%. Internal validation was completed with bootstrap resampling (*n* = 300) in the rms software package in R. Other analyses were performed in SAS 9.3. Details of these analyses can also be found in our prior study.^13^

Analyses of mean value of *EPB41L3* among early and late cases and controls and logistic regression combining oral *EPB41L3* and HPV16 were conducted separately for the newly added cases and controls to examine reproducibility of the biomarker.

## 3 RESULTS

Characteristics of cases and controls are presented in Table 1. The majority of cases (95%) and controls (82%) were white, non‐Hispanic (96% and 88%), and married (79% and 56%). Cases were significantly more likely to have had a tonsillectomy (46% vs. 9.9%) and while matched on smoking status, were more likely to have higher total pack‐years than controls (21% vs. 13% of controls). Cases also had a higher prevalence of any HPV (68% vs. 14%) and HPV16 (54% vs. 3.5%) detected in the oral gargle. Controls were significantly more likely to have 0 (9.9% vs. 3.5% of cases) or 1–9 (30% vs. 23% of cases) lifetime number of people kissed with tongue and have no prior teeth extracted (54% vs. 21% of cases). Among cases, most tumors occurred in the tonsil (51%) or base of the tongue (45%) and most tumors, were positive for HPV of any type (89%) and HPV16 (82%), and rarely HPV18 (4%) positive. There were 92 early disease cases (40.4%) and 136 late disease cases (59.6%), of which 84% and 78%, respectively, were positive for both p16 and HPV in the tumor.

**TABLE 1:** Sociodemographic characteristics of OPC cases compared to controls

Characteristics	Control *N* = 142^a^	Case *N* = 228^a^	*p*‐value^b^
Race			**0.0002**
White	116 (81.7%)	216 (94.7%)	
Black	21 (14.8%)	7 (3.1%)	
Other	4 (2.8%)	5 (2.2%)	
N/A or refused	1 (0.7%)	0 (0%)	
Ethnicity			**0.0226**
Hispanic	16 (11.3%)	9 (3.9%)	
Non‐Hispanic	125 (88.0%)	218 (95.6%)	
N/A or refused	1 (0.7%)	1 (0.4%)	
Median age (years) (SD)	59 (10)	62 (10)	0.3109
Age (years)			0.1168
35–49	22 (15.5%)	22 (9.6%)	
50–59	50 (35.2%)	70 (30.7%)	
60–69	44 (31.0%)	77 (33.8%)	
>=70	25 (17.6%)	59 (25.9%)	
Marital status			**<0.0001**
Married/cohabiting	80 (56.3%)	181 (79.4%)	
Single/divorced/separated/widowed	62 (43.7%)	46 (20.2%)	
N/A or refused	0 (0%)	1 (0.4%)	
Education			0.0508
High school (<12 years)	17 (12.0%)	54 (23.7%)	
Some college/vocational school	56 (39.4%)	80 (35.1%)	
College graduate	41 (28.9%)	62 (27.2%)	
Postgraduate/professional school	28 (19.7%)	31 (13.6%)	
N/A or refused	0 (0%)	1 (0.4%)	
Smoking status			0.2216
Never	57 (40.1%)	103 (45.2%)	
Former	65 (45.8%)	106 (46%)	
Current	20 (14.1%)	18 (7.9%)	
N/A or refused	0 (0%)	1 (0.4%)	
Pack‐years smoking			**0.0130**
Never	57 (40.1%)	106 (46.5%)	
<=5	30 (21.1%)	27 (11.8%)	
6–29	37 (26.1%)	46 (20.2%)	
>=30	18 (12.7%)	49 (21.5%)	
N/A or refused	0 (0%)	0 (0%)	
Alcohol drinks per occasion in the past month			0.3569
None	40 (28.2%)	77 (33.8%)	
1–4	83 (58.5%)	122 (53.5%)	
>=5	18 (12.7%)	26 (11.4%)	
N/A or refused	0 (0%)	3 (1.3%)	
Lifetime number of people kissed with tongue			**0.0181**
None	14 (9.9%)	8 (3.5%)	
1–9	43 (30.3%)	53 (23.2%)	
10–24	33 (23.2%)	62 (27.2%)	
25–49	24 (16.9%)	34 (14.9%)	
>=50	21 (14.8%)	60 (26.3%)	
N/A or refused	7 (4.9%)	11 (4.8%)	
Gave oral sex in past 6 months			**0.0032**
No	75 (52.8%)	121 (53.1%)	
Yes	66 (46.5%)	87 (38.2%)	
N/A or refused	1 (0.7%)	20 (8.8%)	
Tonsillectomy			**<0.0001**
No	128 (90.1%)	122 (53.5%)	
Yes	14 (9.9%)	105 (46.1%)	
N/A or refused	0 (0%)	1 (0.4%)	
Time since tonsillectomy (years ago)			**<0.0001**
Never removed	128 (90.1%)	122 (53.5%)	
<2 years	0 (0%)	23 (10.1%)	
2–29 years	1 (0.7%)	7 (3.1%)	
30+ years	13 (9.2%)	74 (32.5%)	
Gingivitis			0.1683
No	99 (69.7%)	169 (74.1%)	
Yes	38 (26.8%)	57 (25.0%)	
N/A or refused	5 (3.5%)	2 (0.9%)	
Teeth extracted prior to study inclusion			**<0.0001**
0	77 (54.2%)	50 (21.9%)	
1–9	48 (33.8%)	104 (45.6%)	
>10	13 (9.2%)	33 (14.5%)	
N/A or refused	4 (2.8%)	41 (18.0%)	
Tumor Location			
Tonsil		117 (51.3%)	
Base of tongue (BOT)		103 (45.2%)	
Other oropharynx		8 (3.5%)	
Early or late disease presentation			
Early^c^		92 (40.4%)	
Late		136 (59.6%)	
P16ink4a (IHC)			
Positive		198 (86.8%)	
Negative		25 (11%)	
Unknown or N/A		0 (0%)	
Any HPV type (oral)			**<0.0001**
Positive	20 (14.1%)	155 (68.0%)	
Negative	122 (85.9%)	73 (32.0%)	
HPV16 (oral)			**<0.0001**
Positive	5 (3.5%)	122 (53.5%)	
Negative	137 (96.5%)	106 (46.5%)	
HPV18 (oral)			0.0617
Positive	1 (0.7%)	9 (3.9%)	
Negative	141 (99.3%)	219 (96.1%)	
Any HPV type (tumor)			
Positive		203 (89.0%)	
Negative		24 (10.5%)	
HPV16 (tumor)			
Positive		187 (82.0%)	
Negative		40 (17.5%)	
HPV18 (tumor)			
Positive		9 (4.0%)	
Negative		218 (96.0%)	
HR HPV (other than 16/18) (tumor)			
Positive		10 (4.4%)	
Negative		217 (95.6%)	

Bold indicates significant value (*p* < 0.05).

^a^ *n* (%); Median (SD).

^b^ Cochran‐Mantel–Haenszel (Exact).

^c^ Early disease presentation is defined as T1‐2 N0‐1 [small tumors with only a single ipsilateral positive node <3 cm]).

Oral gargle *EPB41L3* methylation was significantly (*p* < 0.0001) higher among cases compared to controls, as well as early disease cases compared to controls (*p* < 0.0004) and late disease compared to controls (*p* < 0.0001). Mean oral gargle *EPB41L3* methylation was highest among late disease cases (3.29 ± 5.61), but mean unadjusted methylation was also higher among early disease cases (1.8 ± 1.55) compared to controls (1.23 ± 0.80). (Table 2). When assessing mean and median *EPB41L3* methylation among newly added cases and controls only, values were only slightly reduced when compared to the combined data (Table S1).

**TABLE 2:** Oral gargle *EPB41L3* methylation status among controls and OPC cases stratified by early (T1‐2 N0‐1 [small tumors with only a single ipsilateral positive node <3 cm]) and late disease presentation

Unadjusted Methylation	Controls *n = 142*	All Cases *n = 228*	Early Disease *n = 92*	Late Disease *n = 136*	*p*‐value^a^
Median	1.14	1.54	1.38	1.57	<0.0001, 0.0004, <0.0001
Mean (SD)	1.23 (0.80)	2.70 (4.50)	1.82 (1.55)	3.29 (5.61)	
IQR (Q1 – Q3)	0.89–1.48	1.17–2.30	1.08–1.95	1.20–3.03	

^a^ *p*‐values are from Wilcoxon Rank Sum tests comparing across two groups, that is, control versus case, control versus early disease and control versus late disease.

**FIGURE 1:**
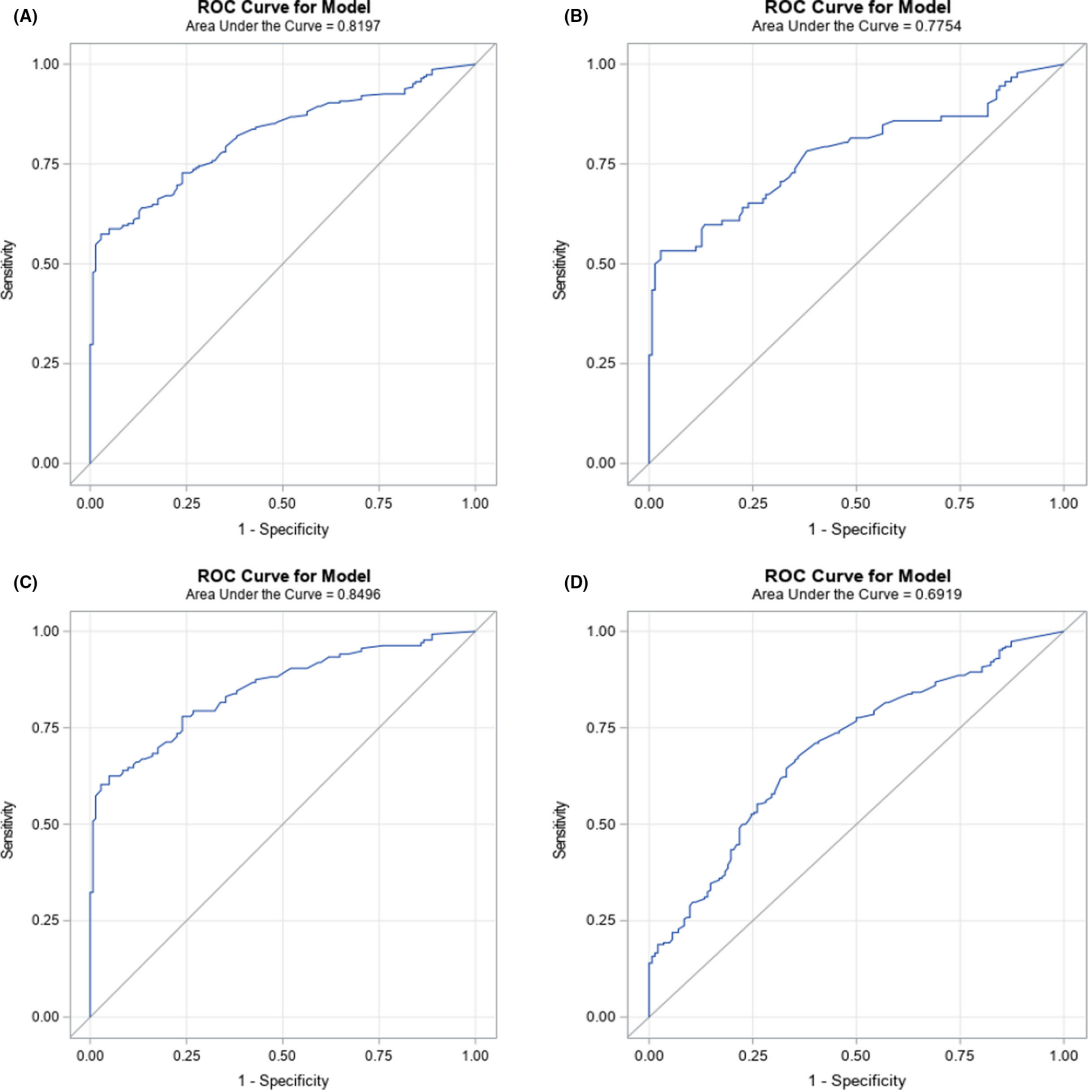
Receiver operating curves showing relationship of sensitivity and 1‐specificity at various cut points based on HPV16 methylation and EPB41L3 methylation to detect oropharyngeal cancer. (A) All cases—ROC was calculated using 142 controls and 228 cases. (B) Early disease cases (T1‐2 and N0‐1, only if there is a single ipsilateral node <3 cm)—ROC was calculated using 92 early cases and 142 controls. (C) Late disease cases–ROC was calculated using 136 late disease cases and 142 controls. (D) ROC using *EPB41L3* methylation alone—ROC was calculated using 228 cases and 142 controls.

ROC curves of the combined oral gargle *EPB41L3*/HPV16 methylation is shown in Figure 1. The ROC for all cases (A), early disease cases (B), and late disease cases (C), along with *EPB41L3* methylation alone (D) illustrate the ability of methylation in each scenario to detect OPC. The area under the curve (AUC) was largest for late stage cases (0.850), followed by all cases (0.820) and early cases (0.775). AUC was lowest for *EPB41L3* methylation alone (0.692) indicating that the addition of HPV16 methylation improved the biomarker's OPC detection ability. When logistic regression was conducted among newly added cases only AUC in all groups changed only slightly (Figure S1).

An optimal specificity indicates a screening test can adequately identify true negative cases and minimize detection of false positives. For rarer outcomes such as OPC, optimal specificity is important. Therefore, biomarker cut points were identified in which specificity remained >70% and sensitivity was also >70%. In both early and late disease, the optimal cut point as identified by Youden's Index (J) was at 3.92. At this score, specificity for both early and late disease was 97%, but at the cost of low sensitivity of 53% (J = 0.504) and 60% (J = 0.575) for early and late disease, respectively. Among late disease cases, decreasing to a score of 1.53 achieved sensitivity of 78% while also maintaining a specificity of 76% (Youden's J = 0.540). However, among early cases, a cut point of 1.22 resulted in a sensitivity of 78% and specificity of 62% (J = 0.402). By increasing the cut point to 1.53, specificity improved to 76%, but sensitivity dropped to 65% (J = 0.413).

## 4 DISCUSSION

We expanded our previous study with the priority to enrich the early OPC case sample size to specifically test the performance of HPV16/*EPB41L3* methylation as a biomarker for early OPC detection. With increased sample size, the results of this study are consistent with our previous study, in that *EPB41L3* methylation alone had marginal test performance (AUC = 0.692), but improved with the addition of HPV16 methylation for all cases (0.820). In this study with additional OPC cases, HPV16 and *EPB41L3* methylation AUC was highest for late disease cases, and was lower among early disease cases compared to the AUC for late disease and all cases. For early disease, gains in specificity for the HPV/host methylation biomarker came at the cost of the biomarker's sensitivity begging the question of whether improvement could be achieved with additional biomarkers.

Methylation of the host tumor suppressor gene, *EPB41L3*, has been shown to be associated with the presence of cancer at the anal canal and cervix.^15^, ^16^ With the addition of the methylation assessment of the HPV16 L1, L2, and E2 genes prediction was extended to also include cervical and anal high‐grade intraepithelial neoplasia, making this a useful screening biomarker for these two HPV‐related cancers.^16^, ^17^ Utilization of this same biomarker panel for HPV‐related OPC could be a useful screening metric for a cancer in which cytological screening is not possible. Our previous study found oral gargle methylation patterns to differ between cases and controls. This study, with a larger sample size, presented a similar pattern of results with AUC highest for late disease, but less robust for the detection early disease compared to healthy controls.

When examining the sensitivity and specificity of HPV and *EPB41L3* methylation for detecting early OPC, high specificity could not be achieved while also maintaining an ideal sensitivity over 70%. At a cut point of 1.53, this was nearly achieved, but specificity could not be achieved greater than 65% without compromising sensitivity. These probabilities, along with an AUC of 0.775 for early disease detection, indicate that the test has some utility, though likely requires additional measures or biomarkers to improve use as a screening test.

Future studies are needed to identify additional markers to develop a more specific and sensitive panel for identifying early OPC. For example, Ren et al identified 20 differentially methylated regions among HPV‐positive OPC cases compared to normal tissues and HPV‐negative OPC cases.^18^ Methods now exist to investigate over 850,000 methylation sites in the human genome, which may lead to the discovery of biomarkers that could have utility in developing a biomarker panel for OPC early detection. Further, other biospecimens have recently been proposed as potential biomarkers of early stage detection of head and neck cancers including circulating tumor cells and extracellular vesicles, both which are often best assessed in salivary samples.^19^

In conclusion, a biomarker panel that detects OPC early, using a specimen that is easily obtained such as the oral gargle, is needed to reduce morbidity and increase survival associated with OPC. Our study has shown the potential for HPV16 and *EPB41L3* methylation as a key factor in early detection of OPC. Additional biomarkers are needed to optimize this panel of methylation markers for early OPC detection.

## AUTHOR CONTRIBUTIONS

All authors have made substantial contributions to the work, given final approval of the manuscript, and have verified the accuracy and integrity of the work. Conception and design was conducted by BLD, BN, and ARG. Acquisition of data was performed by BN, MD, BS, MA, KAIS, KK, CC, and ARG. Analyses of data was performed by MJS, DB, and JW. Interpretation of data was performed by BLD, MJS, and ARG. Manuscript drafting and revising was performed by BLD.

## CONFLICT OF INTEREST

Dr. Giuliano reports grants from Merck & Co, personal fees from Merck & Co outside the submitted work. All other authors report no conflicts.

## ETHICS STATEMENT

Written informed consent was provided prior to study participation. The human subjects’ committees of Advarra Institutional Review Board and the Moffitt Cancer Center Scientific Review Committee provided approval prior to recruitment of OPC cases. For the recruitment of controls from the HIM study, approval was granted from the University of South Florida (United States), Ludwig Institute for Cancer Research (Brazil), Centro de Referencia e Treinamento em Doencas Sexualmente Transmissíveis e AIDS (Brazil), and Instituto Nacional de Salud Publica de Mexico (Mexico).

## Supporting information


Data S1

## Data Availability

The authors confirm that, for approved reasons, some access restrictions apply to the data underlying the findings. Data are available to outside investigators but within the limitations of preserving the anonymity of individuals since these data could theoretically identify a study participant and contain sensitive information. All reasonable data requests should be directed to the Center for Immunization and Infection Research in Cancer Moffitt Cancer Center, Tampa, Florida, 33,612 (email: ciirc@moffitt.org).
