# Supplementary material for: Methylation of HPV16 and EPB41L3 in oral gargles and the detection of early and late oropharyngeal cancer
Source: Cancer Med. 2022 May 26;11(20):3735–42. doi: 10.1002/cam4.4757 (PMC9582688; doi:10.1002/cam4.4757)
Supplement: Supplementary file 1 — Data S1 [file CAM4-11-3735-s001.docx]

# Supplemental Information

**Supplemental Table 1.** Oral gargle *EPB41L3* methylation status among controls and OPC cases of *newly added participants only*, stratified by early (T1-2 N0-1 [small tumors with only a single ipsilateral positive node <3 cm]) and late disease presentation

| **Unadjusted Methylation** | **Controls**  ***n=41*** | **All Cases**  ***n=127*** | **Early Disease**  ***n=73*** | **Late Disease**  ***n=54*** | **P-value**^a^ |
| --- | --- | --- | --- | --- | --- |
| Median | 1.13 | 1.50 | 1.38 | 1.57 | 0.0059, 0.0568, 0.0015 |
| Mean (SD) | 1.24 (0.80) | 2.23 (4.02) | 1.59 (1.24) | 3.10 (5.92) |  |
| IQR (Q1 – Q3) | 0.85-1.50 | 1.09-1.91 | 1.04-1.77 | 1.21-2.25 |  |
| ^a^ P-values are from Wilcoxon Rank Sum tests comparing across two groups i.e. control vs case, control vs early disease and control vs late disease. | | | | | |


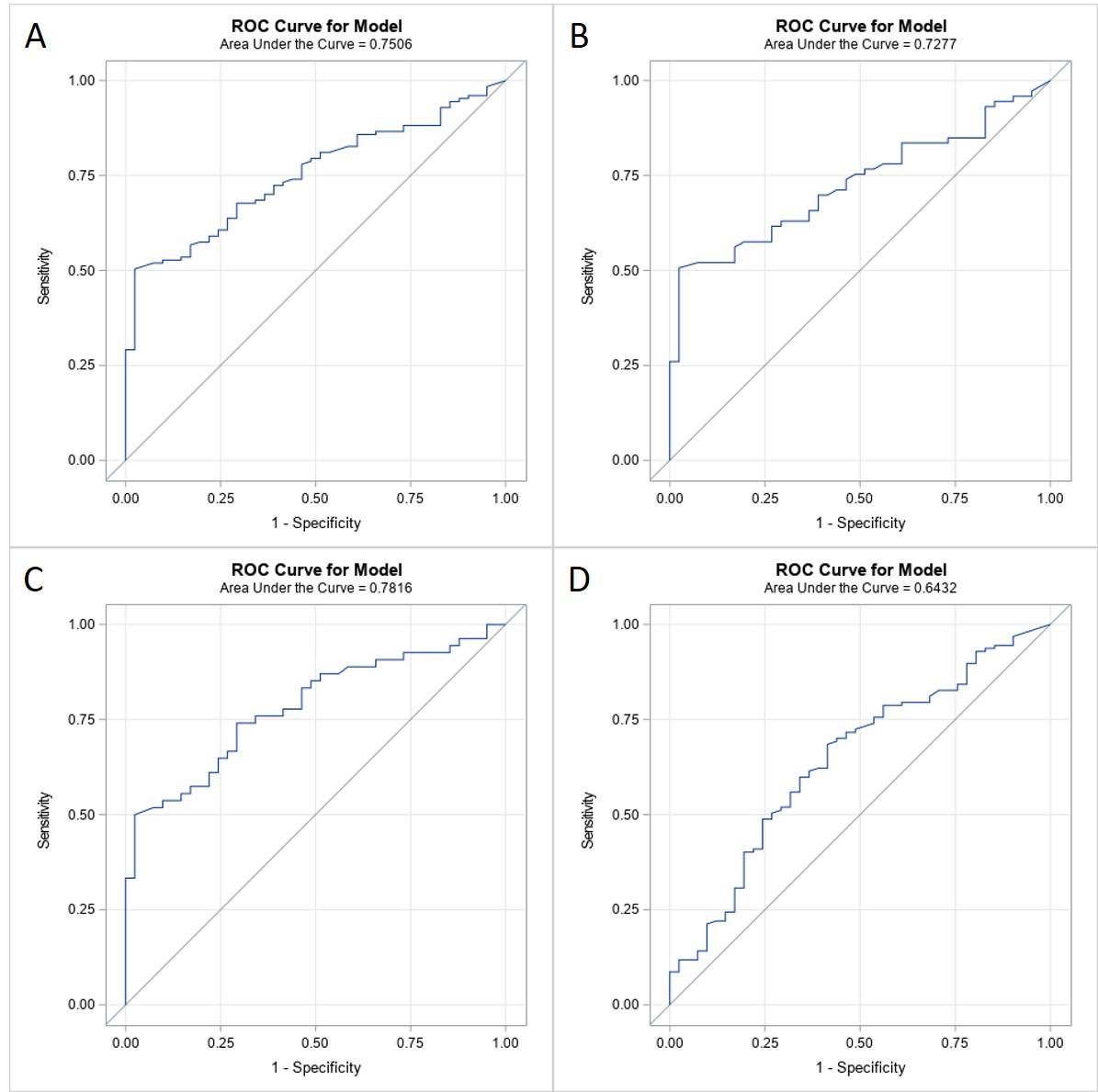


**Supplemental Figure 1.** Receiver operating curves showing relationship of sensitivity and 1-specificity at various cut-points based on HPV16 methylation and EPB41L3 methylation to detect oropharyngeal cancer of *newly added participants only.* A) All cases—ROC was calculated using 41 controls and 127 cases. B) Early disease cases (T1-2 and N0-1, only if there is a single ipsilateral node <3cm)—ROC was calculated using 73 early cases and 41 controls. C) Late disease cases–ROC was calculated using 54 late disease cases and 41 controls. D) ROC using *EPB41L3* methylation alone—ROC was calculated using 127 cases and 41 controls.
